# Bayesian belief network modelling of household food security in rural South Africa

**DOI:** 10.1186/s12889-021-10938-y

**Published:** 2021-05-17

**Authors:** Robert W. Eyre, Thomas House, F. Xavier Gómez-Olivé, Frances E. Griffiths

**Affiliations:** 1 Spectra Analytics, 70 Gracechurch Street, London, EC3V 0HR UK; 2 Department of Mathematics, University of Manchester, Oxford Road, Manchester, M13 9PL UK; 3 Medical Research Council/Wits University Rural Public Health and Health Transitions Research Unit (Agincourt), School of Public Health, Faculty of Health Sciences, University of the Witwatersrand, Johannesburg, South Africa; 4 Warwick Medical School, University of Warwick, Coventry, CV4 7AL UK; 5 University of the Witwatersrand, Johannesburg, South Africa

**Keywords:** Directed acyclic graph, HDSS, Expert elicitation

## Abstract

**Background:**

Achieving food security remains a key challenge for public policy throughout the world. As such, understanding the determinants of food insecurity and the causal relationships between them is an important scientific question. We aim to construct a Bayesian belief network model of food security in rural South Africa to act as a tool for decision support in the design of interventions.

**Methods:**

Here, we use data from the Agincourt Health and Socio-demographic Surveillance System (HDSS) study area, which is close to the Mozambique border in a low-income region of South Africa, together with Bayesian belief network (BBN) methodology to address this question.

**Results:**

We find that a combination of expert elicitation and learning from data produces the most credible set of causal relationships, as well as the greatest predictive performance with 10-fold cross validation resulting in a Briers score 0.0846, information reward of 0.5590, and Bayesian information reward of 0.0057. We report the resulting model as a directed acyclic graph (DAG) that can be used to model the expected effects of complex interventions to improve food security. Applications to sensitivity analyses and interventional simulations show ways the model can be applied as tool for decision support for human experts in deciding on interventions.

**Conclusions:**

The resulting models can form the basis of the iterative generation of a robust causal model of household food security in the Agincourt HDSS study area and in other similar populations.

**Supplementary Information:**

The online version contains supplementary material available at 10.1186/s12889-021-10938-y.

## Background

The Food and Agriculture Organization of the United Nations defines food security as “access of all people at all times to sufficient, nutritionally adequate, and safe food, without undue risk of losing such access” [1]. Many countries have food-insecure populations but globally the number of undernourished people is falling. However, the World Food Summit and the Millennial Development Goal 1.C to halve the proportion of people who suffer from hunger between 1990 and 2015 was not achieved and food insecurity remains an issue for many [2].

Prior to 1999, in South Africa 42% of the population lived below the food poverty line as measured using a quantitative method that relates monthly household food expenses with food insecurity [3]. The number of food insecure people in South Africa decreased between 1999 and 2008 [4] but over a third of children still had low dietary diversity [4] (i.e. few different foods or food groups eaten over a period of time) and the food insecurity rate in rural areas of South Africa is twice that of urban areas [3]. Understanding the determinants of food insecurity therefore remains an important question, even for middle-income countries such as South Africa, particularly in rural areas.

Here we use a methodology that allows us to consider causal interactions between the multiple, interacting variables involved in food security, applied to data from a low resource, rural South African community. In particular, we construct *belief networks* between variables using Bayesian reasoning and consider how these compare with the community’s own beliefs about determinants of food security. Such belief networks can be interpreted, with policy implications drawn out, by non-specialists, and as such we believe that the methodology could usefully be applied more frequently in food security research.

### Study setting

The Agincourt Health and Socio-demographic Surveillance System (HDSS) study area is located in a relatively densely populated low-resource rural setting in rural northeast South Africa close to the Mozambique border. Originally the study area covered 57,600 people in 8900 household and 20 villages [5] but by 2011 it had increased to 90,000 people in 16,000 households and 27 villages [6]. An annual survey of all households has been undertaken since 1992, with full methodological details available elsewhere [5, 6]. The study area is characterized by rudimentary sanitation, poor quality education, and poor quality land that makes agricultural farming difficult, alongside limited healthcare and high unemployment.

Previous studies published on household food security in the Agincourt HDSS study area include, the impacts of adult mortality [7, 8] the impact of the food retail sector [7], the quality of food security indicators [8], and how household food security in the area varied over the 2008 financial crisis [9]. These studies have tended to use standard statistical methods in which e.g. a linear model is used to establish the epidemiological relationship between a proposed determinant and food security.

### Community concerns

Community concern about food security was raised at an MRC/Wits-Agincourt Unit Community Advisory Group (CAG) meeting in 2015. The CAG is composed of individuals who live in the Agincourt HDSS study area and act as liaisons from the community to the research unit. The perception of individuals within the CAG was of food insecurity being a persistent and widespread issue in the area, with high financial constraints, intermittent water access, poor land quality, and the unfashionable status of subsistence farming amongst young people making it difficult to attain enough food each month to live a healthy and active lifestyle. The CAG expressed frustration about not knowing where an intervention might make a difference to food security.

### Modelling interventions in complex systems using Bayesian belief networks

While standard methodology has the benefit that the presence or absence of certain relationships can be hypothesised and statistically tested, this approach is limited in what it is able to tell us about what is clearly a complex system. In particular, we might expect on the basis of expert opinion that there are relationships between the many interrelated characteristics of a household that contribute towards whether the household is or is not food secure. This would mean that a policy based on modification of one determinant might have unexpected consequences due to the effects on other factors. Bayesian belief networks give a graphical representation of the probabilistic dependencies and independencies in a system, presenting a many-to-many view where each variable is taken as random variable which has the potential to have a direct (probabilistic) relationship with any other variable. Although we are interested in food security as an outcome, we can make use of observations of any variables in the model to make inferences relevant to any of the other variables. This is in contrast to the many-to-one view of many other models where we always have one dependent variable being inferred from a group of observed independent variables. The Bayesian belief network approach makes it possible to capture the many and complex relationships expected between variables.

A Bayesian belief network will potentially provide a model of food security with greater ability to capture causal effects and provide a basis for intervention than we might otherwise have. It allows us to reason probabilistically about the system, in particular to ask questions of how each variable impacts on each other variable. Bayesian belief networks encode directional relationships between multiple variables with associated conditional probability distributions. They can then be used to find how the likelihood of one variable having a particular state or value changes given the states or values of other variables in the network through the direct or indirect relationships they have with the variable of interest. This allows us to make interventional, counterfactual, and other more complicated queries of our model [10].

Due to these qualities, Bayesian belief networks have been successfully used in many research areas, including for example semantic search [11], information retrieval [12], analysis of gene expressions [13], medical diagnosis [14], and filtering, smoothing, and prediction [15]. Barons et al. [16] applied the method to food security in the UK. Here we build a Bayesian belief network model with the specific aim of providing a causal representation of the interrelations between various characteristics of households in the Agincourt HDSS study area within the context of food security. Although we aim for the resulting model to provide causal insights into the system, we note that validating the accuracy of any causal relationships the model implies is difficult, and it is best viewed as a tool for decision support by human experts.

In subsequent sections, we give details of the construction of our specific Bayesian network from choosing the variables, through learning the structure and parameters, to seeking quantitative validation of the model. Finally, we give some elementary applications of the networks in interventional inferences.

## Methods

### Formal description of Bayesian belief networks

As discussed in the Introduction above, Bayesian networks are often used to give a representation of a set of causal beliefs about the system, often initially obtained from experts. We define our model as a finite set of random variables $\mathcal{V}$, which are also the vertices of a network, together with a set of edges $\mathcal{E}\subset \mathcal{V}\times \mathcal{V}$. A link from variable *A* to variable *B* (mathematically, $\left(A,B\right)\in \mathcal{E}$) implies that *A* causes *B*, as well as that *B* is directly probabilistically dependent on *A*.

Each of the possible states of each variable in the network are encoded through conditional probability tables (CPTs) for each variable. An entry in the CPT of a variable contains the probability that the variable is in a particular state given its parents are each in particular states of their own. For example, if the food security variable had one direct parent, such as socio-economic status, then the CPT for food security would contain probabilities that the household is food insecure or food secure given socio-economic status is high, that it is food insecure or food secure given socio-economic status is low, and so on. Which variables are linked to which other variables forms the discrete structure of our model, and the entries of the conditional probability tables are the continuous parameters of our model.

While a full algorithmic description of Bayesian belief networks is beyond the scope of this paper, many excellent resources exist on details of the topic [17–19]. For inferences performed on our data with completed network models, we obtained good performance from the use of the Lauritzen-Spiegelhalter (LS) algorithm [20], as implemented by the gRain package in the software R [21]. This algorithm is a specialised variation of belief propagation, taking advantage of the network structure to eliminate variables and simplify the calculation of probability distributions when making inferences on the likelihood of variables having a particular value given the values other variables in the network [20].

### Overview of model building process

**Fig. 1:**
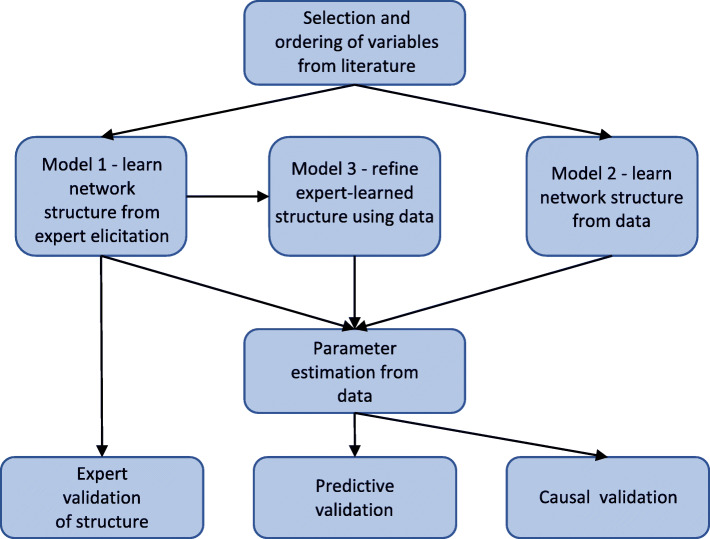
Process for building models. First variables are selected and ordered via literature search. Then three separate network structures are learned from expert elicitation only, data only, and data with the expert network as a prior. Parameter estimation and appropriate model validations are performed for all three networks

Here we provide a summary overview of the process undertaken to build the Bayesian belief network model. Further detail for each step is given in subsequent subsections. The entire process is illustrated in Fig. 1.

Given the initial motivation for this study came from the MRC/Wits-Agincourt Unit Community Advisory Group (CAG), as discussed in the Introduction, we would expect that using expert knowledge from members of the community would help with learning the structure of the network. This introduces a strong element of community involvement in the work and, on the assumption that such expert knowledge contains useful information about the system, it can improve the possibility of discovering causal structure, which is difficult to achieve when learning from data alone [22].

For expert elicitation to work, there must be no latent variables not covered in the variables presented to experts. We kept this in mind when selecting variables. When performing the expert elicitation, we followed a protocol stated in [22], designed specifically for eliciting a causal structure from expert knowledge.

Whilst expert elicitation can maximise the chances of learning a causal structure, it relies entirely on the knowledge of the experts, which could be narrowed by their life experiences. Our experts were community members rather than experts who had studied the problem as used by Barons et al. [16]. We therefore compared expert elicitation for learning network structure with learning the structure algorithmically from the data.

We were also able to construct a causal network combining both the expert results and data-based learning by using the expert network as a prior for algorithms to build on using the data.

The model building process (Fig. 1) therefore involved the following steps:

1. Select variables and their order in the network models (i.e. which variables are considered possibly causally dependent on which other variables) via a literature search [22].
2. Given the variables selected, specify our dataset.
3. Construct the structure of our first network model via expert elicitation.
4. Construct the second network model structure by learning from data.
5. Using the expert elicited network structure as a prior, use data to learn a refined structure as the third network model.
6. For each network use parameter estimation to calculate the conditional probability tables (CPTs).
7. Validate the models using causal validation, as well as a predictive validation and, for the expert elicited model, expert validation of the structure.

### Variable selection and ordering

The first step in building our Bayesian belief networks, as detailed by the protocol being followed, involves a literature search to discover the variables [22].

The first step involved searching the literature for household characteristics that form possible causes of change in household food security, which is our utility variable. The second step then involved searching the literature for household characteristics that form possible causes of change in the characteristics found in step one. The third step looked for characteristics that form possible causes of those from step two, and so on. This was repeated for as many steps as were possible, subject to two constraints. First, we could only select variables that are measured in the Agincourt HDSS dataset. Secondly, we needed to limit the burden on the community experts when eliciting the structure of the network. We therefore limited the variables to those of greatest importance and relevance to food security. Further details of the literature search, the resulting variables in each level and how each variable was calculated, can be found in the Electronic Supplementary Material.

Breaking up the variables into levels gave us a causal ordering of the variables. This ordering is important when eliciting the network structure from experts and it helps minimize the possibility of leaving out latent variables that could confound any causal findings.

### Variable state calculation and specifying the dataset

Most of the variables were calculated using a suitable combination of variables in the Agincourt HDSS dataset, calculated at or aggregated to the household level. While most variables can be straightforwardly taken from the data, two require additional discussion.

The first such variable is Food Security, which was calculated from a single variable in the Agincourt HDSS dataset specifying whether the household had enough food to eat over the past year. While definitions of food security in the literature are often more nuanced than this measure, most other variables related to food security in the Agincourt HDSS dataset feature large amounts of missing data, but this variable does not. It is also strongly correlated with many more subtle measures of food security, despite its simplicity. When considering the results of applying the completed Bayesian network, however, we must simply remember that food security in this case is defined as having had enough food to eat over the past year.

The second such variable is the local vegetation level. Following Nawrotzki et al., we calculated this from the Normalised Difference Vegetation Index (NDVI) [9, 23]. NDVI data was obtained from the MODIS/Terra sensor satellite images, which contains NDVI values for 250 m areas averaged over day periods [24]. As per Nawrotzki et al., local vegetation level is calculated for a particular household as the NDVI over the 2000 km region around the household (not including land within villages so as to avoid including privately owned land) averaged over the households within that region. Though Nawrotzki et al. averaged these values over the preceding 3 years of the year of analysis, we only averaged them over the year of analysis to avoid removing detail of differences between each year.

Variables that were calculated from the Agincourt HDSS food security module were done so for the years 2007, 2010, and 2013, i.e. the years in which the module was performed during the census. For the other variables, their value from the nearest previous year when the data to calculate them was collected was used for each household at each of the three food security module years. Our sample was formed by households that provided complete enough data to calculate values for each of the variables for at least one of the food security module years. For each household, the latest entry from either 2007, 2010, or 2013 was taken, as it was found that allowing households to have multiple entries for different years reduced the performances of the models seen in the model validations and comparisons. The sample size for our dataset was 11,739 households.

Although it would be possible to construct a dynamic Bayesian network with nodes for the different variables at each of the different time points [19], the data was only sufficiently informative to support construction of a static network.

Values/states of each variable were taken from the values/states recorded in the Agincourt HDSS questions. Several of the variables were discretised, as detailed in the Electronic Supplementary Material, in order to reduce the number of possible states and simplify the calculations involved in finding the parameters of the model and performing inferences. This was done in such a way to coarse-grain the distribution of the variable, either by combining neighbouring states that occurred rarely, or by binning the data for that variable to an appropriate number of histogram bins.

### Structure learning via expert elicitation

We performed an expert elicitation to find a possible causal structure for the Agincourt HDSS food security belief network, relying on the knowledge of members of the Community Advisory Group (CAG). To perform this elicitation we relied on the protocol described in [22]. After selecting a set of variables and a causal ordering for them, as described above, we carried out the elicitation. A pilot study for the elicitation was carried out on a convenience sample of University students and employees (including from a non-mathematical background) to confirm that the process would be understandable for experts.

The elicitation was performed by the Head of the Public Engagement Office of the MRC/Wits-Agincourt Unit following our design and instructions in the MRC/Wits Agincourt Unit offices on 28th October 2016. The experts were guided through a list of the variables, in the order defined by the causal ordering starting with the highest level variables (which our ordering specifies have no possible causes within the other variables, but are possible causes for all the other variables) and finishing with the lowest level variable food security (which is specified to cause none of the other variables, and can be caused by all other variables). For each variable, we attempted to establish based on the local knowledge of our experts which of the previous variables in the list would have an effect on the beliefs of the experts on what state the variable in question would take for a generic household. We carefully designed our questions to attempt to capture the conditional independences between the variables by asking the experts to first consider the hypothetical situation where they already know the states for a household of all the variables previous in the list except one, and then whether learning the state of that one extra variable would provide any extra impact on their belief of the variable of interest.

In order to prepare the experts for the elicitation, they were first told the purpose of the work and what outcomes are hoped for, as well as a brief lay explanation of Bayesian networks. They were guided through a set of example questions on a different smaller system taken from the Agincourt HDSS study area in order to help them understand how to answer the questions, and to get them used to answering them before having to answer the ones we cared about. The elicitation was performed with the experts as a group to reduce the burden. The experts were also reassured that none of their answers would be incorrect in order to avoid any biases such as adjusting their answers to try and get to the ‘right one’.

### Structure learning via data

In order to learn an alternative possible causal network for our system we used a constraint-based algorithm known as Max-Min Parents and Children (MMPC) [25, 26]. For each node A, the MMPC algorithm attempts to discover the set of parents and children of A. The MMPC algorithm can be performed for each node to find all the local structures and construct a skeleton of the network (i.e. with no directions).

This was performed for our Agincourt HDSS household food security dataset using the asymptotic normal Jonckheere-Terpstra test for conditional independence to take into account the ordinal nature of the variables [27]. Links that would go against the causal ordering we defined in our literature search were disallowed from the start. The order of the variables used in the expert elicitation was used to gain directions in the skeleton network achieved by the algorithm. The algorithm was trained on all 11,739 households in the dataset, each household acting as an individual observation.

### Parameter estimation

Formally speaking, the network parameters for the model are:

$$ {\uptheta_i}^{jk}=\Pr \left({A}_i=j\;|\; pa\left({A}_i\right)=k\right), $$

where *A*_*i*_ is an individual variable, *j* is a possible state of that variable, *pa*(*A*_*i*_) are the parent variables of *A*_*i*_ in the network, and *k* are the states of the parent variables. Although it is possible to elicit these from experts as well, we instead estimated them from data. This both avoided the many cognitive biases that the experts could be prone to, and avoided placing an additional and immense burden on the experts on top of what we have already asked of them. The parameters were estimated by their maximum a posteriori (MAP) values found by maximising the product of the multinomial likelihood of the network (arising from the discrete nature of all the variables) with a Dirichlet prior that was equivalent to add-one smoothing [17]. The choice of prior eases computations as Dirichlet is the conjugate prior of the multinomial likelihood. The add-one smoothing adjusts for cases where no occurrences of a particular combination of variable and parent states appear in the data. Like with using the data to learn the network structure, the parameters were fitted using all 11,739 households, with each acting as an individual observation.

### Expert validation

Conditional and unconditional independences in a Bayesian network can be found using the mathematical concept of d-separation [19, 22]. We performed one validation of our expert elicited structure by testing relationships found via d-separation with the expectations of our experts.

Since over 8000 different independence relationships are implied by the network’s structure, the number to be worked with was severely reduced in a structured fashion. In order to make it simpler for the experts, only relationships of the kind *A* ⊥ *B* ∣ *C* where *A* and *B* only contained one variable each and *B* came before *A* in the causal ordering defined in the literature search were questioned. From these, examples where the fact that *A* is at all independent of *B* appears counter-intuitive were selected as these seemed the most pertinent to check. Finally, for each possible pair of *A* and *B* in the remaining relationships, the relationship *A* ⊥ *B* ∣ *C* where *C* contained the smallest set of nodes was selected, again to make it easier for the experts. This resulted in 34 relationships to check against the beliefs of the experts.

This elicitation was performed with a new set of experts, still taken from the CAG. It was again performed by Head of the Public Engagement Office of the MRC/Wits-Agincourt Unit following our design and instructions, located in the MRC/Wits-Agincourt Unit offices in March 2017.

### Predictive validation

The ability of a model to predict does not imply that it is a good causal model. Nevertheless, predictive power is a desirable feature of a causal model, and so we test for it.

To achieve this, we performed a 10-fold cross validation for each network, where the network structures were kept constant but parameter values were allowed to vary dependent on the data subset they were being fitted to. Since there are different classifiers to allow us to best distinguish between the networks. The first, Briers scoring, gives a heavier penalisation the lower the predicted probability of the correct food security state of the household, with severe penalisation given for particularly extreme incorrect results [28]. The second, information reward, prefers models that estimate probabilities of the correct state that are better than random and penalize ones that are worse [29]. The last, Bayesian information reward, prefers models that estimate probabilities of the correct states that are better than some chosen prior probabilities (here chosen to be equal to the empirical frequency of food insecure households in the training data), and then penalises ones that are worse – thereby indicating whether a given network structure allows for better prediction of food insecurity than what we can tell by simply looking at the data [30].

### Causal validation

There is no widely accepted way to measure the faithfulness of a model to the causal relationships of the system it represents using observational data. Rather, the accepted method for validation of causal structure is to measure the effects of interventions on the outcomes of interest. Since running our own interventions would have been impractical and unethical, we looked for data on food security interventions performed on similar populations to ours, and looked to see which models (if any) successfully capture these results. To do this we performed a thorough search of the literature, looking for interventions aimed at food security in populations similar to that of the Agincourt HDSS study area i.e. rural populations in low to middle income countries throughout Sub-Saharan Africa, Asia, and Latin America. We then performed interventional inferences examining the effect of such interventions on the probability of a household being food insecure. This was done using the standard approach necessary for interventional inferences where the state of the interventional variable is set in the network and all incoming links to that variable are removed before calculating the conditional probability of food insecurity given the interventional variable being in the chosen state. This reflects the fact that in this case the variable has been artificially set by an outside force, and is no longer dependent on the factors that usually influence it [19].

### Applications

Finally, we considered some applications of such a Bayesian network model for food security. One interesting application is in using them to find which of the variables have the greatest probabilistic impact on food security, which can be done through a sensitivity analysis. This was performed following Barons et al. by calculating values for mutual information reduction and expected change of belief [16].

The most useful application of a causal Bayesian network is in simulating possible interventions on it to see whether they should be attempted in actuality. As such, we performed some possible novel interventional strategies based on findings from a search of the literature for food security interventions in rural populations of low to middle income countries.

## Results

### Network structure

**Fig. 2:**
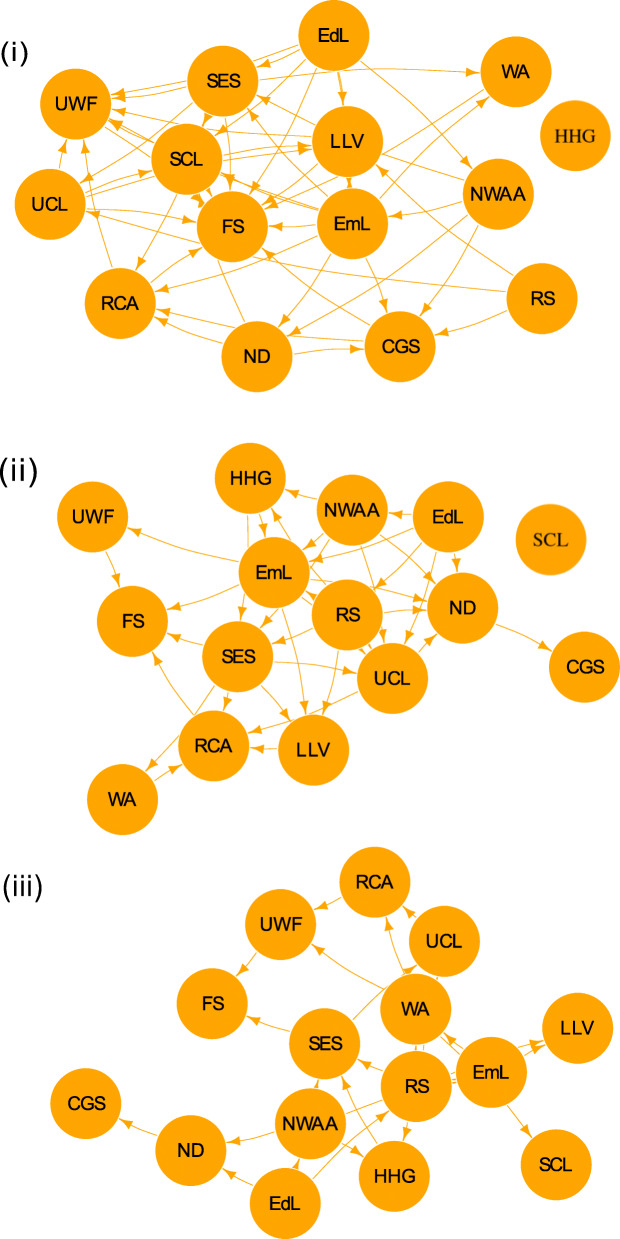
Agincourt HDSS food security belief networks with structure learned from (i) expert elicitation only (ii) data only (iii) data with the expert network as a prior. CGS – child grant status. EdL – education level. EmL – employment level. FS – food security. HHG – household head gender. LLV – level o3f3 local vegetation. ND – number of dependents. NWAA – number of working age adults. RCA – receipt of communal aid. RS – refugee status. SCL – selling of crops and livestock. SES – socio-economic status. UCL – use of crops and livestock. UWF – use of wild foods. WA – water access

The variable-selection step of the expert elicitation resulted in the inclusion of the following variables alongside food security, further details of which can be seen in the Electronic Supplementary Material: child grant status (CGS); education level (EdL); employment level (EmL); household head gender (HHG); level of local vegetation (LLV); number of dependants (ND); number of working age adults (NWAA); receipt of communal aid (RCA); refugee status (RS); selling of crops and livestock (SCL); socio-economic status (SES); use of crops and livestock (UCL); use of wild foods (UWF); and water access (WA). The resulting network structures for each of our three possible methods of structure learning can be seen in Fig. 2. The figure only show the network structures and not the accompanying conditional probability tables that parameterise the models due to the very high number of parameter values in the model (the combination of all possible states and all possible parent states of each variable). As we are interested in the possible causal implications of the models, given by the structures themselves, this is still of value.

The resulting network from the expert elicitation (Fig. 2(i)) shows some interesting discrepancies from findings in the literature. For instance, expert opinion is that household head gender has no impact on any of the other variables. Refugee status is also held to have minimal direct impact. Also, water access is elicited as having no impact on the growing of crops due to individuals relying on rainwater rather than the water supply to water their crops and gardens.

The data-learned network (Fig. 2(ii)) shows some substantial differences compared to the expert elicited network, which is unsurprising given the very different sets of information each network was built upon. Most different is the much greater level of sparseness in the data-learned network. Household head gender is also linked into the data-learned network, unlike the expert elicited one, but selling of crops and livestock is now disconnected. Child grant status has no directed path to food security either, meaning that neither of these nodes would be effective means of intervention according to this network.

The final resulting network from a combination of expert and data learning (Fig. 2(iii)) appears sparser than the other two but still has all nodes linked into the network. There are also more leaf nodes in the network with no children such as selling of crops and livestock, level of local vegetation, and child grant status again. If this approach is correct, these nodes would be ineffective for any interventions against food insecurity.

Despite the efforts to ensure relationships going against causality were avoided, some seemingly counter-intuitive ones still appeared such as the number of working age adults and refugee status having a causal impact on household head gender. These relationships are not, however, counter-intuitive when interpreted correctly – e.g. the gender of an individual is not determined by refugee status, but refugee status can influence which gender heads the household.

### Model validation results

The results of the initial expert-based validation of the expert structure implies that the initial elicited structure proves quite robust against checks of relationships discovered via d-separation. Only 11 out of 34 of the relationships were deemed to be false (though 2 were unanswered). Typically this was due to the new set of experts thinking that household head gender does impact on certain variables, Mozambican refugees are now indistinguishable from South African nationals, and that both age and education impact on attitudes which in turn impact on things such as the willingness to farm, forage for food, and claim welfare.

When it came to the predictive validation, unsurprisingly learning the structure from data gave greater predictive performance than relying on the expert elicitation in terms of overall cross validation scores (Table 1). The data gave a greater population view of what is happening than the experts, although did not give an improvement on performance compared to simply relying on the empirical data frequencies. Using data-learning with the expert network as a prior results in an increase in performance over the data-learned method and in fact is the only method to improve upon relying on the empirical frequencies.

**Table 1:** Model comparison of predictive performance for the different network structures learned from a 10-fold cross validation. Briers score, information reward, and Bayesian information reward indicate that learning from data massively outperforms learning from experts, but using expert knowledge as a prior provides an additional small increase in performance

Learning method	*S*_**B**_	*S*IR	*S*BIR
Expert elicitation	0.1321	0.3908	−1.9004
Data-learned	0.0851	0.5577	−0.0270
Data-learned with expert prior	0.0846	0.5590	0.0057

Examining the confusion matrices, calculated by learning the models from a randomly chosen subset forming 90% of the data and then predicting the food security state of the remaining 10%, shows less of a distinction between the different models (Table 2). However, using data-learning with the expert network as a prior still seems to give some improvement here. The state of the confusion matrices is also reliant on the random splitting of the data used. Due to the use of cross validation, the combination of scores still give a more reliable comparative measure of performance between the models.

**Table 2:** Predictive confusion matrices for each network structure, displaying numbers of true positives, false positives, true negatives, and false negatives. These values were calculated by learning the structure and parameter values from a random subset forming 90% of the overall data, then making predictions for whether a household is food secure or not for the remaining 10% of the data. These show less distinction between the different network structures in terms of performance but using expert knowledge as a prior still seems to give a small boost

	True state	
Predicted state	Food secure	Food insecure
(a) Expert elicitation		
Food secure	570	34
Food insecure	492	79
(b) Data-learned		
Food secure	554	42
Food insecure	508	71
(c) Data-learned with expert prior		
Food secure	617	44
Food insecure	445	69

### Model simulations and comparison with intervention studies

The literature review for real food security interventions performed in rural populations in low to middle income countries found that several agricultural interventions have been performed in the form of community agricultural projects, homestead food production, and the development of gardens in countries and regions such as Bangladesh, South-East Asia, Latin America, Rwanda, South Africa itself, and other low to middle income countries [31–37]. Various financial interventions have also been performed, such as providing public sector employment and cash transfers in Latin America and Ethiopia [35, 38], as well as micro-loans and micro-credit in sub-Saharan Africa [39, 40]. In addition to these, we also found interventions on water quality throughout Asia and Africa [34].

It should be noted that whilst some of the interventions discovered by the literature review were parts of controlled studies, others were reports of government strategies that therefore may not have successfully controlled for confounding variables which may undermine any causal implications we can take from them. They may also be subject to publication bias, causal structure may vary across space and time meaning that we cannot compare across years and countries, and some consider a different definition of food security from ours. Nevertheless, it is interesting to look at what our networks would predict for interventions analogous to those in the empirical studies.

**Fig. 3:**
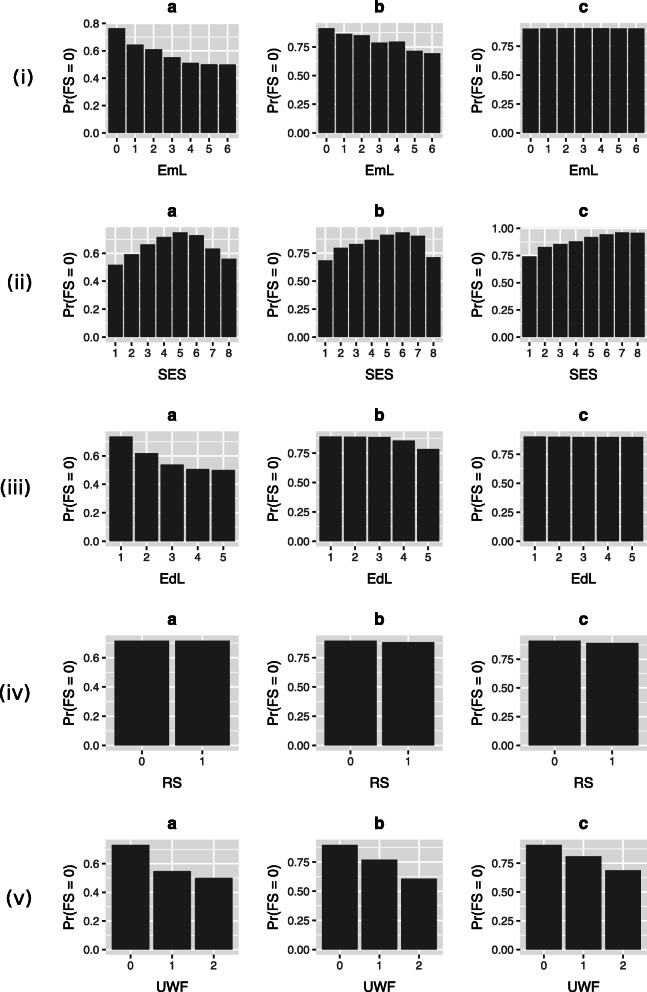
Single intervention predictions. Simulations of setting the state of (i) ‘employment level’ (EmL) (ii) ‘socio-economic status’ (SES) (iii) ‘education level’ (EdL) (iv) ‘refugee status’ (RS) (v) ‘use of wild foods’ (UWF) in order to alter32 the probability of a household being food secure (Pr(FS = 0)) on the different possible Agincourt HDSS food security belief networks. **a**: expert elicited structure; **b**: data-learned structure. **c**: data-learned structure with the expert network as a prior

**Fig. 4:**
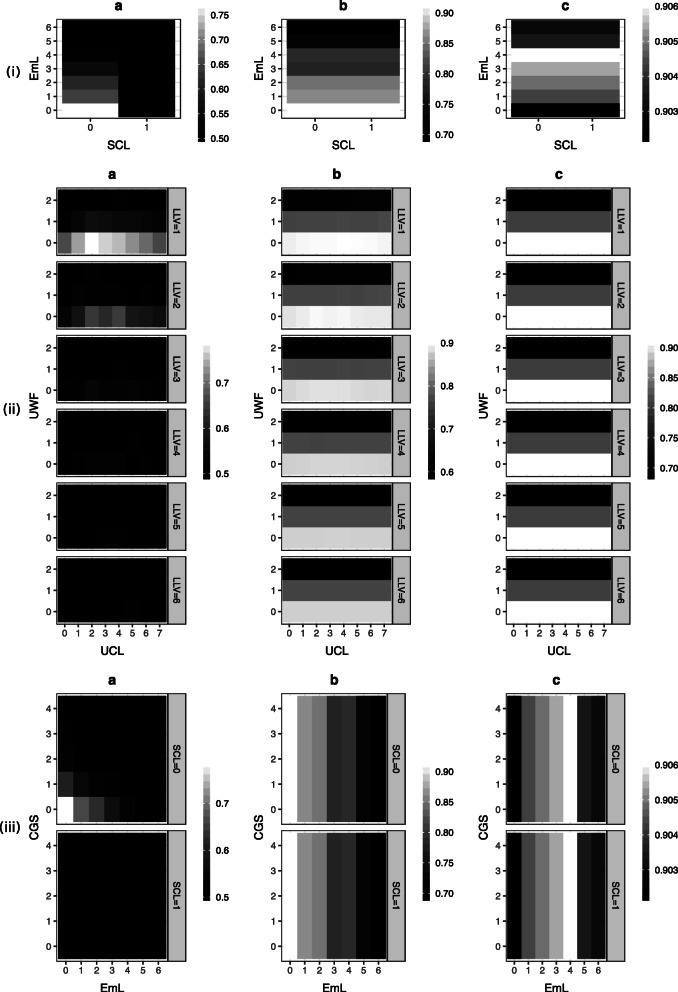
Multiple / complex intervention predictions. Interventional inference on the impact of (i) both ‘employment level’ (EmL) and ‘selling of crops and livestock’ (SCL) simultaneously (ii) ‘level of local vegetation’ (LLV), ‘use of wild foods’ (UWF), and ‘use of crops and livestock’ (UCL) (iii) ‘child grant status’ (CGS), ‘employment level’ (EmL), and ‘selling of crops and livestock’ (SCL) on the probability of a household being food secure (Pr(FS = 0)) on the different possible Agincourt HDSS food security belief networks. **a**: expert elicited structure; **b**: data-learned structure. **c**: data-learned structure with the expert network

The interventions found in the literature therefore gave us possible simulations to run on our networks. In particular, we simulated setting the values for the ‘use of crops and livestock’, ‘child grant status’, ‘selling of crops and livestock’, ‘socio-economic status’, ‘employment level’, and ‘water access’ variables individually and looked at the impact on the probability of the household being food secure. The simulation results can be seen in Figs. 3 and 4, with further results in the Electronic Supplementary Material, and we see from these that food security changes depend on both the variable(s) changed and the causal network structure.

In particular, we see that for employment and education levels (Fig. 3(i, iii)) the expert elicited and data-learned networks produce counter-intuitive results – i.e. that increases to these variables decrease the probability of food security – that conflict with empirical studies, while the network combining data learning with expert elicited priors produces results that do not. For socio-economic status (Fig. 3(ii)), we see consistently strong effects, which implies that socio-economic status is as important to food security as we expect it to be, perhaps more so than any other household characteristic. Given how it relates so heavily to many household characteristics it is not surprising that it could capture much of what makes a household food secure or not. For this variable, the monotone response of the network combining data learning with expert elicited priors is more consistent with intuition and empirical observations.

We stress that the considerations of confounding, publication bias, context etc. above mean that these results cannot provide more than very indirect evidence in favour of one causal network or another. They do, however, show that much more could be done to validate different causal representations of the system, although this is beyond the scope of this current work. Nevertheless, it is suggestive that the combination of data learning and expert elicitation is more consonant with other sources of information than either data or elicitation alone.

### Applications

Our results indicate possible avenues for interventional studies if the networks are taken with a causal interpretation. Otherwise, they show us the variables with the greatest probabilistic relationships with food security (Table 3), which may aid in streamlining efforts to identify potential food insecure households, which may be helpful given how rarely food security information is collected compared to other information. The expert elicited network results also give an indication of what variables have the greatest impact on the beliefs of a member of the community when considering what other households may be food insecure.

**Table 3:** Sensitivity analysis results

	a: Expert elicitation			b: Data-learned			c: Data + expert prior		
Variable	*I*	*I*/*H*	_*S*_2	*I*	*I*/*H*	_*S*_2	*I*	*I*/*H*	*S*^2^
CGS	0.0099	0.0115	0.0030	0.0000	0.0000	0.0000	0.0002	0.0004	0.0000
EdL	0.0078	0.0091	0.0023	0.0000	0.0000	0.0000	0.0001	0.0001	0.0000
EmL	0.0177	0.0205	0.0052	0.0001	0.0002	0.0000	0.0082	0.0165	0.0013
HHG	0.0000	0.0000	0.0000	0.0002	0.0004	0.0000	0.0000	0.0001	0.0000
LLV	0.0264	0.0307	0.0079	0.0000	0.0000	0.0000	0.0028	0.0056	0.0005
ND	0.0021	0.0025	0.0006	0.0001	0.0001	0.0000	0.0008	0.0016	0.0001
NWAA	0.0007	0.0008	0.0002	0.0007	0.0014	0.0001	0.0005	0.0011	0.0001
RCA	0.0083	0.0097	0.0025	0.0000	0.0001	0.0000	0.0066	0.0133	0.0010
RS	0.0000	0.0000	0.0000	0.0007	0.0016	0.0001	0.0003	0.0005	0.0000
SCL	0.0108	0.0126	0.0034	0.0000	0.0000	0.0000	0.0000	0.0000	0.0000
SES	0.0067	0.0078	0.0020	0.0112	0.0244	0.0014	0.0114	0.0228	0.0017
UCL	0.0039	0.0046	0.0011	0.0011	0.0025	0.0001	0.0008	0.0017	0.0001
UWF	0.0334	0.0388	0.0101	0.0043	0.0093	0.0007	0.0066	0.0133	0.0012
WA	0.0049	0.0056	0.0014	0.0000	0.0000	0.0000	0.0001	0.0003	0.0000

Mutual information reduction *I* (also divided by food security information *H*) and expected change of belief *S*^*2*^ for food security given each of the other variables for the three different networks

*CGS* child grant status, *EdL* education level, *EmL* employment level, *FS* food security, *HHG* household head gender, *LLV* level of local vegetation, *ND* number of dependents, *NWAA* number of working age adults, *RCA* receipt of communal aid, *RS* refugee status, *SCL* selling of crops and livestock, *SES* socio-economic status, *UCL* use of crops and livestock, *UWF* use of wild foods, *WA* water access

When considering possible interventions for similar populations (rural populations in low to middle income countries), various observational studies imply that education, communal aid, local vegetation, and being a refugee should each individually have a substantial effect on food security [9, 41, 42]. However, our inferences imply that these variables actually have only negative, if any, impact on food security (examples for education level and refugee status are shown in Fig. 3(iii) and (iv), with further results available in the Electronic Supplementary Material) dependent on the network used. This only matches the literature for refugee status, where it implies we should expect a negative impact (although our effect is quite small).

Dovie et al. found that use of wild foods is prevalent in the Bushbuckridge district, within which the Agincourt HDSS study area is situated [43]. Performing an intervention on this variable shows that increased use of wild foods leads to lower probability of food security (Fig. 3(v)) completely irrespective of the network. It is possible here that the direction of the relationship is incorrect, as perhaps more food insecure households use foraging as a coping strategy so that use of wild foods only occurs after food insecurity has been attained.

Mabuza et al. found that non-farming income provides for greater food security than farming income [44]. If we look at a combined inference of selling of crops and livestock and employment level at the same time (Fig. 4(i)) we find that selling crops and livestocks makes largely no difference. Controlling for it though does allow for an impact from employment level, though this is only positive for the data-learned network with the expert prior where the effect is also quite small.

M’Kaibi et al. examined the impact of both agriculture and the environment on food security [45]. We can do the same by performing a combined inference on level of local vegetation, use of wild foods, and use of crops and livestock (Fig. 4(ii)). Both vegetation level and use of crops and livestock have little to no impact but holding them fixed again allows an impact from use of crops and livestock (though not so much for the expert elicited network). As we have seen before, this impact is still negative for both data-learned networks which perhaps lends greater support to our earlier hypothesis of the relational direction between food security and use of wild foods being opposite to that in the models.

Finally, Pereira et al. examined the impact of income on food security [7]. We can look at different income sources by performing a combined inference on selling of crops and livestock, child grant status, and employment level (Fig. 4(iii)). As expected from the sensitivity analysis, child grant status and selling of crops and livestock have little impact. Therefore, we conclude that actual income is more important to food security than welfare or commercial farming, though it is only a positive impact for the data-learned network with expert prior again. This implies that it is perhaps the paths going through these other nodes in the network that leads to the switching of the relationship between employment and food security to being negative.

## Discussion

In this paper we have considered the building of three different potential causal Bayesian belief network models of household food security in rural South Africa. Other research in this area has often been performed using generalised linear models, statistical tests, and summary statistics [7–9, 46, 47]. This has produced many worthwhile results, but there are limitations with what can be inferred from, and what actions can be justified by, these models. The Bayesian network models we presented here better reflect the complexity inherent within the system. This is achieved by the inbuilt modelling of indirect probabilistic relationships. In this way we end up with more of a ‘many-to-many’ model rather than the ‘many-to-one’ model that linear regression gives us. Accompanied by a causal explanation, this enables us to more effectively model different possible actions, events, and interventions on the system.

On top of this better reflection of complexity, network models also come with a higher level of interpretability. They better reflect what we see in the world around us, where many things cannot be simply considered as having direct and linear relationships with each other. The resulting models are therefore much more satisfying in both their utility and their ability to represent the system.

Though we were unable to test the causal faithfulness of our models to the system, this is a problem that is shared by all the methods that came before. Our models are still much more suitable for causally modelling the system, as they have the directional structure that is necessary in order to do so [48]. The lack of such structure undermines the ability to make any causal inferences from the models of previous studies. Time could have been incorporated into the model by designing a dynamic Bayesian network, but doing so would have severely limited the amount of data that could be used to estimate each parameter as well as requiring the uncertain assumption that causal effects last over 3 years due to the gap between collection of the food security census module. It would have also presented a much greater additional burden to our experts in the elicitation, which we were expressly unable to do due to our limited resources.

If the causal interpretation of any one of the three possible household food security models presented here can be confirmed, then that model will be a valuable tool in designing and simulating basic interventions against food insecurity in the Agincourt HDSS study area. In particular, the model can be evolved via an iterative process of using it to inform intervention trials and then using the results of these trials to further develop the model. Though food insecurity is decreasing in sub-Saharan Africa [4, 49] it is still a great concern of the Agincourt HDSS community, as discovered by our meetings with the MRC/Wits-Agincourt Unit Community Advisory Group. Demonstrating actions and improvements in this area would obviously be of great importance to them, and may therefore further reinforce their support of the research unit (though support is already great due to the benefits the census already provides the area). Beyond the Agincourt HDSS study area, the models could also form the basis for the design of similar models in similar populations across the world.

As with all research, this study comes with limitations. The MRC/Wits-Agincourt Unit goes to great efforts to ensure the reliability of the Agincourt HDSS data [6].

Despite this, there are some errors, misreporting, and missing data that we are unable to account for. However, the dataset is of a size and quality that these do not produce any substantial issues and do not seriously undermine the results presented or the completeness of the data in terms of its ability to cover all variables needed to fully causally model the system without the need of any latent variables. This means that again the choice of variables is a great limitation as it is possible that latent variables may have been missed, though this was again mitigated by choosing the variables in an informed way.

In addition, the results from the expert elicitation produced some very counter intuitive findings. The findings may be heavily related to the nature of the experts we used, as our experts were experts in life in the locality. Other studies, such as that of Barons et al., used experts that had a greater overview of a wide range of evidence on the topic in question and great understanding of that evidence [16]. The benefits of such an approach must be traded off against community involvement in research, but it raises the interesting possible future research area as to the effect of using different expert groups. Though the results we obtained from our expert elicitations are quite possibly biased by the locality of their knowledge, the community-driven nature of that particular analysis can still be viewed as a great advantage. It is important in Public Health, considering the complexity of the systems analysed, the public nature of research funding, and the possibility of biases in our research due to lack of knowledge or perspective, to involve the individuals who our research is based on into that research.

Overall, these limitations do not undermine the usefulness of developing these models, though do emphasise the need to perform more research with much greater resources before the models can be fully put into practise to model interventions.

## Conclusions

We believe that the Bayesian belief network models of household food security we presented here can form the basis of the iterative generation of a robust causal model of household food security in the Agincourt HDSS study area and elsewhere. As there is no established test of causal faithfulness beyond experimentation, the models can be used to inform possible interventional studies that could then be used to further develop the model and so on. In addition, it is possible that methods of testing causal faithfulness from observational data could be explored, but this comes with obvious limitations. An alternative to experimentation would be to use further expert elicitations. Another way to improve the causal applicability of the methods would be to develop them into dynamic Bayesian networks that include temporal relationships, which would also require further resources in terms of data and expert elicitations. Beyond the further development of these models, they can also form the basis of developing similar models of household food security for other populations.

## Supplementary Information

**Additional file 1.** Bayesian belief network modelling of household food security in rural South Africa - supplementary material. Further details on the variable selection literature search, as well as further results for both the simulations of established food security interventions and the interventional inferences.

## Data Availability

The data that support the findings of this study are available from the Medical Research Council/Wits University Rural Public Health and Health Transitions Research Unit but restrictions apply to the availability of these data, which were used under license for the current study, and so are not publicly available. Data are however available from the authors upon reasonable request and with permission of the Medical Research Council/Wits University Rural Public Health and Health Transitions Research Unit. The MOD13Q1: MODIS/Terra Vegetation Indices 16-Day L3 Global 250 m SIN Grid V006 data was retrieved from the online Data Pool, courtesy of the NASA EOSDIS Land Processes Distributed Active Archive Center (LP DAAC), USGS/Earth Resources Observation and Science (EROS) Center, Sioux Falls, South Dakota, 10.5067/modis/mod13q1.006.

## Abbreviations

HDSS: Health and socio-demographic surveillance system
BBN: Bayesian belief network
DAG: Directed acyclic graph
CAG: Community advisory group
CPT: Conditional probability table
LS: Lauritzen-Spiegelhalter algorithm
NDVI: Normalised difference vegetation index
MMPC: Max-min parents and children
BIC: Bayesian information criterion
MAP: Maximum a posteriori
CGS: Child grant status
EdL: Education level
EmL: Employment level
HHG: Household head gender
LLV: Level of local vegetation
ND: Number of dependants
NWAA: Number of working age adults
RCA: Receipt of communal aid
RS: Refugee status
SCL: Selling of crops and livestock
SES: Socio-economic status
UCL: Use of crops and livestock
UWF: Use of wild foods
WA: Water access
