# Supplementary material for: Bayesian belief network modelling of household food security in rural South Africa
Source: BMC Public Health. 2021 May 17;21:935. doi: 10.1186/s12889-021-10938-y (PMC8130258; doi:10.1186/s12889-021-10938-y)
Supplement: Supplementary file 1 — Additional file 1. Bayesian belief network modelling of household food security in rural South Africa - supplementary material. Further details on the variable selection literature search, as well as further results for both the simulations of established food security interventions and the interventional inferences. [file 12889_2021_10938_MOESM1_ESM.pdf]

Bayesian belief network modelling of household food security in rural South Africa - supplementary material.

R W Eyre

T House

F X Gómez-Olivé

F E Griffiths

Contents

1 Variable Selection Literature Search 1

2 Further results for simulations of established food security interventions 10

3 Further results for interventional inferences 12

1 Variable Selection Literature Search

This section details the relationships found within the literature that informed the construction of the Agincourt food security Bayesian belief networks.

Household food security in the Agincourt study area was found in the literature to be influenced by whether households participate in subsistence or commercial farming,

forage for food from the local environment, receive aid from the community, or receive government child welfare grants; as well as on their socio-economic status, how well educated the household members are, what the gender of the household head is, how many individuals in the household are too young or old to work, and how many working age adults live in the household (defined as aged 15-59 as individuals make their greatest economic contribution to the household from the age of 15 [1], and the age at which individuals in South Africa can claim an older persons grant is 60 [2]) [1, 3–9]. Whether a household forages for food from the environment was found to be influenced by the numbers of individuals in the household too young or old to work, as well as the number at working age [10], and is obviously also affected by the level of local vegetation in the local area. Whether a household is likely to receive communal aid (e.g. food or money) was found to be influenced by their socio-economic status, the gender of their household head, and whether the household contains refugees (i.e. individuals not defined in the dataset as South African nationals, usually due to being Mozambican refugees) [7, 11]. Households selling crops and livestock is obviously influenced by whether they grow or rear them in the first place. Whether households claim child support grants was found to be influenced by how well employed the household members are and their socio-economic status (which is unsurprising as the grant is means tested [12]), as well as their refugee status and obviously by whether the household has any children [6,7,13]. Whether the household grows crops and livestock was found to be influenced by their socio-economic status, how good their access to water is, and whether they have enough household members to perform the farming [3, 6]. Household socio-economic status was found to be influenced by the household levels of employment and education, as well as their refugee status and the gender of their household head [6].

These relationships gave us a causal ordering of the different household variables, as shown by the breaking up into levels of the variables in Tables 1 to 9. The variables of each level can only be caused by a variable in a higher numbered level, and can only cause those in a lower numbered level, according to this ordering. For example, water access in level 5 (Table 5) is possibly a cause of variables such as use of

crops and livestock, use of wild foods, and food security itself which are all in lower numbered levels, but is disallowed from causing variables such as socio-economic status, refugee status, or education level which are in higher numbered levels. This ordering was important when attempting to ensure a causal structure when eliciting the network structure from experts.

Variable	Definition	States	Data source
Food insecure	Whether the household has or has not had enough to eat over the previous year.	0 - had enough to eat.<br>1 - did not have enough to eat.	NotEatYear variable in the Agincourt Food Security module.

Table 1: Agincourt food security belief network variables on level 1 of the causal ordering. The data source specifies how the variable is calculated from the data, and the states specify how the discrete variable states relate to the values resulted from the calculations described in the data source.

Variable	Definition	States	Data source
Use of wild foods	Whether the household uses wild foods.	0 - do not use wild foods.<br>1 - gather wild foods OR wild herbs.<br>2 - gather wild foods AND wild herbs.	Sum of the SupGather and SupGatherHerbs variables from the Agincourt Food Security module.
Receipt of communal aid	Whether the household gets financial aids from friends, neighbours, or family.	0 - receive no aid.<br>1 - receive aid.	SupDonate variable from the Agincourt Food Security module.

Table 2: Agincourt food security belief network variables on level 2 of the causal ordering. The data source specifies how the variable is calculated from the data, and the states specify how the discrete variable states relate to the values resulted from the calculations described in the data source.

Variable	Definition	States	Data source
Local vegetation level	Amount of vegetation that grows in the local area of the household.	1 - up to 0.161.<br>2 - 0.161-0.302.<br>3 - 0.302-0.443.<br>4 - 0.443-0.584.<br>5 - 0.584-0.725.<br>6 - greater than 0.725.	Sum of the average NDVI values from the MODIS Terra satellite data in a 2000km by 2000km area around the household divided by the number of households in that area.
Selling of crops and livestock	Whether the household sells crops or livestock to supplement their income.	0 - sell no crops or livestock.<br>1 - sell crops or livestock.	Sum of the SupSellGoods and SupSellStock variables from the Agincourt Food Security module.
Child grant status	Number of child grants the household receives.	State is equal to number of grants received that year.	Count of grants received using the ReceivedYear variable from the Agincourt Child Grant module.

Table 3: Agincourt food security belief network variables on level 3 of the causal ordering. The data source specifies how the variable is calculated from the data, and the states specify how the discrete variable states relate to the values resulted from the calculations described in the data source. Value intervals are inclusive of the upper values.

Variable	Definition	States	Data source
Number of dependents	Number of household members aged less than 15 or greater than 59.	1 - up to 9.5.<br>2 - 9.5-19.0.<br>3 - 19.0-28.5.<br>4 - 28.5-38.0.<br>5 - 38.0-47.5.<br>6 - greater than 47.5.	Count of household members (membership counted at midyear) with the appropriate age (calculated at midyear) in the Agincourt dataset.
Use of crops and livestock	Whether the household grows its own crops or keeps its own livestock.	0-7+.	Sum of the MaizePlot, MaizeField, OtherCropsPlot, and OtherCropsField variables from the Agincourt Food Security module with the Cattle, Goats, Poultry, and Pigs variables from the Agincourt Asset Status module.

Table 4: Agincourt food security belief network variables on level 4 of the causal ordering. The data source specifies how the variable is calculated from the data, and the states specify how the discrete variable states relate to the values resulted from the calculations described in the data source. Value intervals are inclusive of the upper values.

Variable	Definition	States	Data source
Water access	Availability, stability, and quality of the water supply to the household.	1 - up to 10.5.<br>2 - 10.5-16.0.<br>3 - 16.0-21.5.<br>4 - 21.5-27.0.<br>5 - 27.0-32.5.<br>6 - 32.5-38.0.<br>7 - 38.0-43.5.<br>8 - greater than 43.5.	Product of the WaterDistMetre, WaterAvail, and WaterSup variables from the Agincourt Asset Status module.

Table 5: Agincourt food security belief network variables on level 5 of the causal ordering. The data source specifies how the variable is calculated from the data, and the states specify how the discrete variable states relate to the values resulted from the calculations described in the data source. Value intervals are inclusive of the upper values.

Variable	Definition	States	Data source
Socio-economic status	Socio-economic status of the household.	1 - up to 1.262.<br>2 - 1.262-1.648.<br>3 - 1.648-2.033.<br>4 - 2.033-2.419.<br>5 - 2.419-2.804.<br>6 - 2.804-3.190.<br>7 - 3.190-3.575.<br>8 - greater than 3.575.	SESAbsolute variable from the Agincourt SES index module.

Table 6: Agincourt food security belief network variables on level 6 of the causal ordering. The data source specifies how the variable is calculated from the data, and the states specify how the discrete variable states relate to the values resulted from the calculations described in the data source. Value intervals are inclusive of the upper values.

Variable	Definition	States	Data source
Employment level	Number of household members with current employment.	0-6+ employed household members.	Count of household members (membership counted at midyear) who replied yes to the CurrentlyWorking variable in the Agincourt Labour Status module.
Household head gender	Gender of the head of the household.	0 - male.<br>1 - female.	Household head gender as specified in the Agincourt dataset.
Refugee status	Whether any household members are refugees or non-South African nationals.	0 - no refugee members.<br>1 - one or more refugee members.	Refugee variable from the Agincourt dataset.

Table 7: Level Agincourt food security belief network variables on level 7 of the causal ordering. The data source specifies how the variable is calculated from the data, and the states specify how the discrete variable states relate to the values resulted from the calculations described in the data source.

Variable	Definition	States	Data source
Number of working age adults	Number of household members aged 15 to 59.	1 - up to 13.29.<br>2 - 13.29-26.57.<br>3 - 26.57-39.86.<br>4 - 39.86-53.14.<br>5 - 53.14-66.43.<br>6 - 66.43-79.71.<br>7 - greater than 79.71.	Count of household members (membership counted at midyear) with appropriate age (calculated at midyear) from the Agincourt dataset.

Table 8: Agincourt food security belief network variables on level 8 of the causal ordering. The data source specifies how the variable is calculated from the data, and the states specify how the discrete variable states relate to the values resulted from the calculations described in the data source. Value intervals are inclusive of the upper values.

Variable	Definition	States	Data source
Education level	Average years of completed education by the household members.	1 - up to 3.<br>2 - 3-6.<br>3 - 6-9.<br>4 - 9-12.<br>5 - greater than 12.	Average of the EducationInYears variable from the Agincourt Education module for household members (membership counted at midyear) for that year.

Table 9: Agincourt food security belief network variables on level 9 of the causal ordering. The data source specifies how the variable is calculated from the data, and the states specify how the discrete variable states relate to the values resulted from the calculations described in the data source. Value intervals are inclusive of the upper values.

2 Further results for simulations of established food security interventions

Figures 1 to 4 show further results for the simulations performed to test the three different network structures on their ability to capture established interventions from the literature.

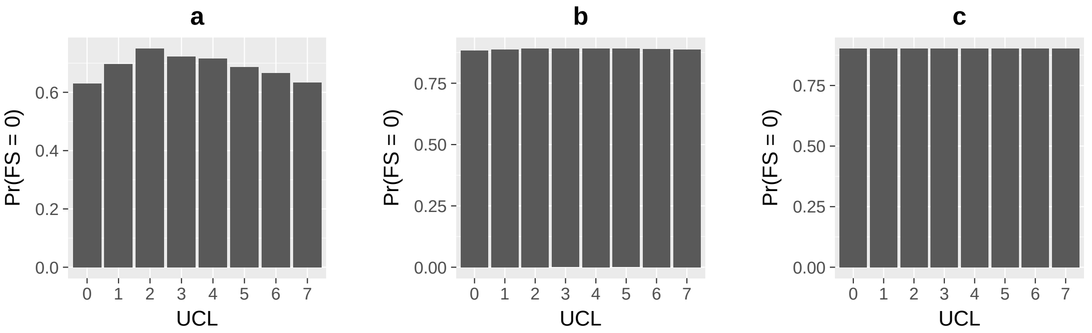

Figure 1: Simulations of setting the state of 'use of crops and livestock' (UCL) in order to alter the probability of a household being food secure ($FS = 0$) on the different possible Agincourt food security belief networks. a - expert elicited structure. b - data-learned structure. c - data-learned structure with the expert network as a prior.

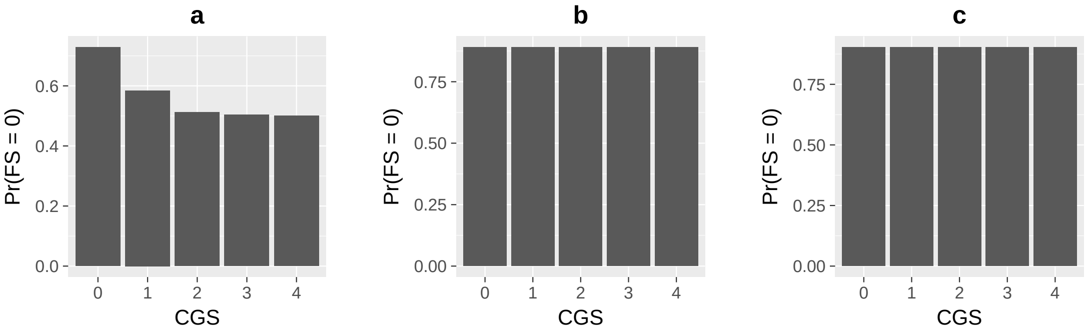

Figure 2: Simulations of setting the state of 'child grant status' (CGS) in order to alter the probability of a household being food secure ($FS = 0$) on the different possible Agincourt food security belief networks. a - expert elicited structure. b - data-learned structure. c - data-learned structure with the expert network as a prior.

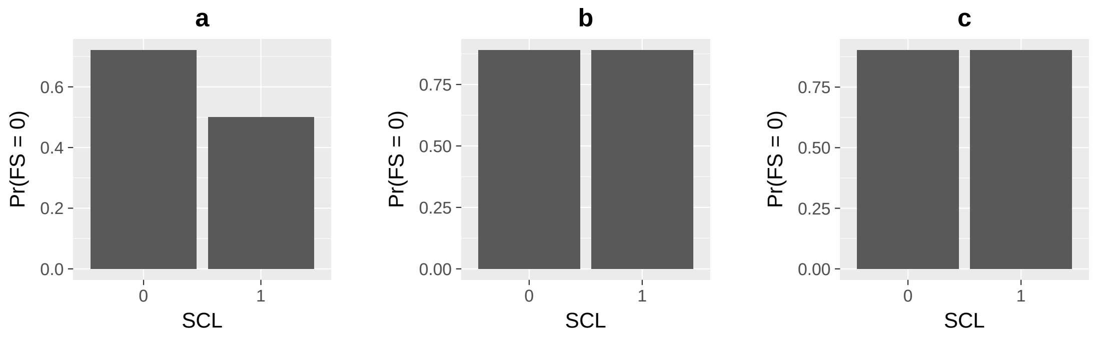

Figure 3: Simulations of setting the state of 'selling of crops and livestock' (SCL) in order to alter the probability of a household being food secure ($FS = 0$) on the different possible Agincourt food security belief networks. a - expert elicited structure. b - data-learned structure. c - data-learned structure with the expert network as a prior.

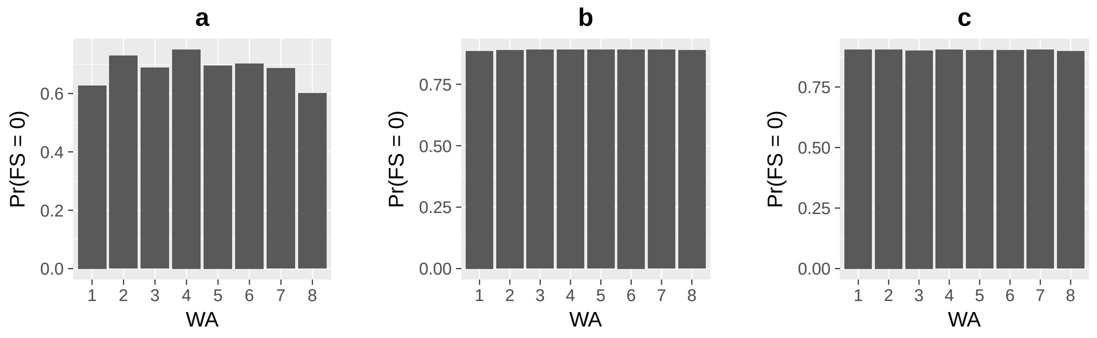

Figure 4: Simulations of setting the state of 'water access' (WA) in order to alter the probability of a household being food secure ($FS = 0$) on the different possible Agincourt food security belief networks. a - expert elicited structure. b - data-learned structure. c - data-learned structure with the expert network as a prior.

3 Further results for interventional inferences

Figures 5 and 6 show further results for the interventional inferences performed on the three different network structures to demonstrate applications of the models.

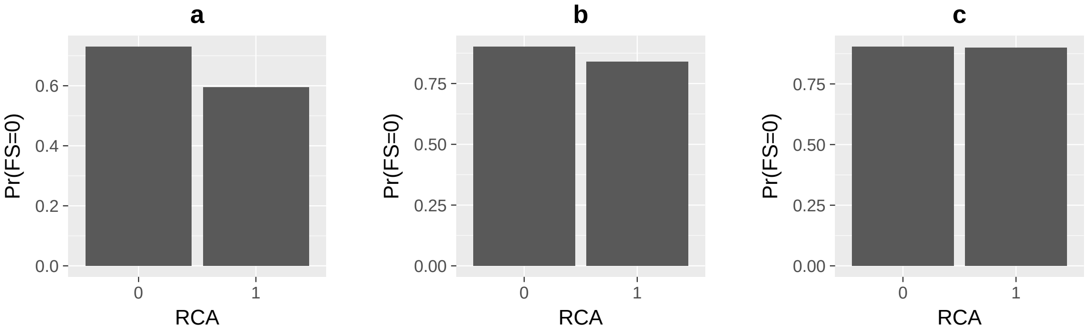

Figure 5: Interventional inference on the impact of 'receipt of communal aid' (RCA) on the probability of being food secure (Pr (FS = 0)). a - expert elicited network. b - data-learned network. c - data-learned network with the expert network as a prior. Depending on the network, child grants have either a negative impact or none at all on food security.

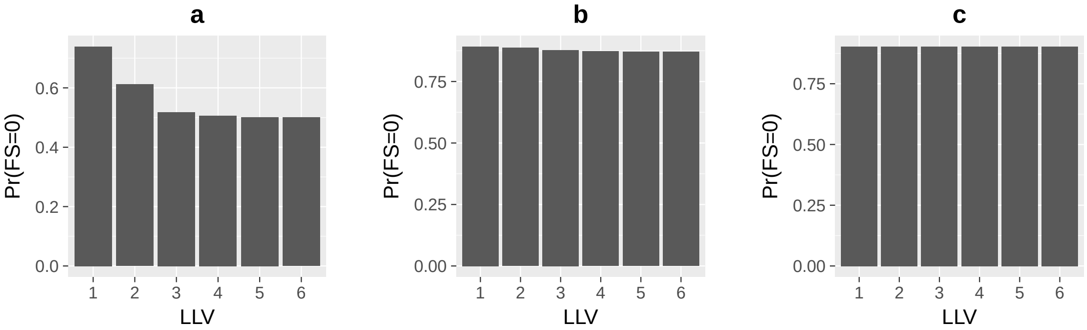

Figure 6: Interventional inference on the impact of 'level of local vegetation' (LLV) on the probability of being food secure (Pr (FS = 0)). a - expert elicited network. b - data-learned network. c - data-learned network with the expert network as a prior. Depending on the network, local vegetation has either a negative impact or none at all on food security.

References

[1] Twine W, Hunter LM. Adult mortality and household food security in rural South Africa: Does AIDS represent a unique mortality shock? Development Southern Africa. 2011;28(4):431–444.

[2] South African Social Security Agency. Grants for Older Persons; 2017. Accessed: 27 October 2017. http://www.sassa.gov.za/index.php/social-grants/grants-for-older-persons.

[3] Hunter LM, Twine W, Patterson L. "Locusts are now our beef": Adult mortality and household dietary use of local environmental resources in rural South Africa. Scandinavian Journal of Public Health. 2007;35(69_suppl):165–174.

[4] Kirkland TM, Kemp RJ, Hunter LM, Twine WM. Toward improved understanding of food security: a methodological examination based in rural South Africa. Food, Culture & Society. 2013;16(1):65–84.

[5] Nawrotzki RJ, Robson K, Gutilla MJ, Hunter LM, Twine W, Norlund P. Exploring the impact of the 2008 global food crisis on food security among vulnerable households in rural South Africa. Food security. 2014;6(2):283–297.

[6] Pereira LM, Cuneo CN, Twine WC. Food and cash: understanding the role of the retail sector in rural food security in South Africa. Food security. 2014;6(3):339–357.

[7] Kaschula S. Using people to cope with the hunger: social networks and food transfers amongst HIV/AIDS afflicted households in KwaZulu-Natal, South Africa. AIDS and Behavior. 2011;15(7):1490.

[8] Lemke S, Vorster HH, van Rensburg NJ, Ziche J. Empowered women, social networks and the contribution of qualitative research: broadening our understanding of underlying causes for food and nutrition insecurity. Public Health Nutrition. 2003;6(8):759–764.

[9] Sedibe HM, Kahn K, Edin K, Gitau T, Ivarsson A, Norris SA. Qualitative study exploring healthy eating practices and physical activity among adolescent girls in rural South Africa. BMC Pediatrics. 2014;14(1):211.

[10] Dovie DB, Shackleton CM, Witkowski E. Conceptualizing the human use of wild edible herbs for conservation in South African communal areas. Journal of Environmental Management. 2007;84(2):146–156.

[11] Pronyk PM, Harpham T, Busza J, Phetla G, Morison LA, Hargreaves JR, et al. Can social capital be intentionally generated? A randomized trial from rural South Africa. Social Science & Medicine. 2008;67(10):1559–1570.

[12] South African Social Security Agency. Child Support Grant; 2017. Accessed: 27 October 2017. http://www.sassa.gov.za/index.php/social-grants/child-support-grant.

[13] Twine R, Collinson MA, Polzer TJ, Kahn K. Evaluating access to a child-oriented poverty alleviation intervention in rural South Africa. Scand J Pub Health. 2007;35(69_suppl):118–127.
